# Identification of Type 2 Diabetes-associated combination of SNPs using Support Vector Machine

**DOI:** 10.1186/1471-2156-11-26

**Published:** 2010-04-23

**Authors:** Hyo-Jeong Ban, Jee Yeon Heo, Kyung-Soo Oh, Keun-Joon Park

**Affiliations:** 1 Division of Bio-Medical Informatics, Center for Genome Science, National Institute of Health, Korea Center for Disease Control and Prevention, 194, Tongil-Lo, Eunpyung-Gu, Seoul 122-701, Republic of Korea

## Abstract

**Background:**

Type 2 diabetes mellitus (T2D), a metabolic disorder characterized by insulin resistance and relative insulin deficiency, is a complex disease of major public health importance. Its incidence is rapidly increasing in the developed countries. Complex diseases are caused by interactions between multiple genes and environmental factors. Most association studies aim to identify individual susceptibility single markers using a simple disease model. Recent studies are trying to estimate the effects of multiple genes and multi-locus in genome-wide association. However, estimating the effects of association is very difficult. We aim to assess the rules for classifying diseased and normal subjects by evaluating potential gene-gene interactions in the same or distinct biological pathways.

**Results:**

We analyzed the importance of gene-gene interactions in T2D susceptibility by investigating 408 single nucleotide polymorphisms (SNPs) in 87 genes involved in major T2D-related pathways in 462 T2D patients and 456 healthy controls from the Korean cohort studies. We evaluated the support vector machine (SVM) method to differentiate between cases and controls using SNP information in a 10-fold cross-validation test. We achieved a 65.3% prediction rate with a combination of 14 SNPs in 12 genes by using the radial basis function (RBF)-kernel SVM. Similarly, we investigated subpopulation data sets of men and women and identified different SNP combinations with the prediction rates of 70.9% and 70.6%, respectively. As the high-throughput technology for genome-wide SNPs improves, it is likely that a much higher prediction rate with biologically more interesting combination of SNPs can be acquired by using this method.

**Conclusions:**

Support Vector Machine based feature selection method in this research found novel association between combinations of SNPs and T2D in a Korean population.

## Background

It is estimated that by the year 2030, there will be ~366 million people affected by Type 2 diabetes (T2D) worldwide [1], with many of those affected lying in the middle to late adult years group [2]. T2D is genetically heterogeneous disease by the complex interplay of several environmental factors and susceptibility genes [3]. Single-nucleotide polymorphism (SNP) exhibits an abundant form of genetic variations. SNPs can be distinguished from other rare variations by more than 1% frequency in the human population when a single nucleotide replaces one of the three nucleotides. The human genome contains about 10~30 million SNPs with an average SNP every 100~300 bases. More than 5 million human SNPs have been identified and the information is publicly available (NCBI dbSNP Build 129). A SNP in a protein coding sequence (CDS) can induce amino acid changes, resulting in functional changes in the protein. Some SNPs in a promoter region can effect transcriptional regulation, and a SNP in an intron region can affect the splicing or expression of the gene.

In recent years, genome-wide association studies (GWAS) have identified a large number of robust associations between genetic variation and complex human disease, such as Type 2 diabetes and rheumatoid arthritis [4]. These approaches have identified common genetic variants that are associated with the risk of more than 40 diseases and human phenotypes [5]. In the T2D studies, candidate gene or genome-wide association approaches have suggested various putative T2D susceptibility SNP variants in various genes including *TCF7L2*, *PPARG*, *KCNJ11*, *CDKN2A/B*, *FTO, CDKAL1 *and so on [6-10]. But individual susceptibility of SNP variants may be disappointingly small or nowhere near enough to explain estimates of heritability [11]. One possible explanation for these weak relative risks and low attributable risks is that the risk may vary across different groups of clinically and biologically distinct T2D; further, analyzing T2D as a single disease may obscure the association with these risk factors. Another possible explanation is the effects of gene-gene (SNP-SNP) interactions. Most complex diseases result from the poorly understood interaction of genetic-genetic and genetic-environmental factors. The biological phenomenon associated with T2D that are modestly affected by a single SNP might be much greatly affected by a SNP in combination with additional SNPs in genes derived from the same or distinct biological pathways. In other words, it is difficult to identify disease-linked variants that are too rare to be picked up by association methods and yet have risk alleles of sufficient effects to allow detection with the use of existing statistical strategies [12]. A marker strongly related to risk does not guarantee effective discrimination between cases and controls [13].

A goal of this research is to assess the rules for classifying the case (T2D) and control (non-T2D) groups along with considering the potential gene-gene (SNP-SNP) interactions. Since it is considered that the SNPs are less influential toward the onset or development of T2D than combinations of SNPs, our interest is specially focused on the classification of SNPs that can detect the putative effects of genetic interactions. Small effects that could, when combined, have a significant impact on someone's health including onset of T2D, thus to get overall view of risk, the effects of the individual SNPs have to be combined [11]. There are several researches designed to examine the effect of combined SNPs to disease risks. Some methods have used the multifactor dimensionality reduction (MDR) algorithm, which identifies all the possible combinations of SNPs from a set of given SNPs, and the combination of SNPs that optimally predicts the risk by minimizing the classification error of cases and controls is finally selected [14]. Goodman and colleagues formulated a polymorphism interaction analysis (PIA) method, which examines all the possible SNP combinations (similar to MDR) among 94 SNPs in 63 genes studied in 216 male colon cancer cases and 255 male controls. They employed two separate functions that cross-validate and minimize the false-positive results in the evaluation of SNP combinations to predict the risk of colon cancer [15].

In the present research, we analyzed the associations between the combination of SNPs and T2D using a Support Vector Machine (SVM) - a machine learning algorithm. Classification based on the SVM has several applications in bioinformatics and computational biology [16-20]. It has been widely used to predict protein secondary structures [21], solvent accessibility [22,23], protein-protein binding sites [24], remote protein homology detection [25], detection of non-coding RNA [26], protein domains [27], protein subcellular localization [28-30], discrimination of outer membrane protein [31], and gene and tissue classification from the microarray expression data [32].

Several researchers have recently applied this powerful machine-learning algorithm--SVM--to the problem of identifying combinations of SNPs that can predict the susceptibility toward diseases. Listgarten and colleagues [33] considered the SNPs from 45 genes of potential relevance to breast cancer etiology in 174 patients as compared to the matched normal controls. They obtained an accuracy of 69% when using SVMs as the learning algorithm. They concluded that multiple SNPs from different genes over distant parts of the genome are better at identifying breast cancer patients than any single SNP alone. Waddell *et al*. (2005) have applied SVMs to predict the susceptibility to multiple myeloma. Their work provided 71% accuracy on a dataset containing 40 cases and 40 controls. Very recently, Uhmn *et al*. (2009) applied several machine learning techniques including SVM to predict patients' susceptibility to chronic hepatitis from SNPs [34].

In this research, we analyzed the importance of gene-gene interactions on T2D risk by investigating 408 SNPs from 87 genes involved in major T2D-related pathways in a sample of 462 T2D cases and 456 healthy population controls. We applied the SVM to discriminate cases and controls with SNP combination information by means of a 10-fold cross-validation test. From the target population, we achieved 65.3% prediction rate with a combination of 14 SNPs from 12 genes using the RBF (Radial Basis Function) kernel SVM. We also investigated men and women sub-population datasets using the same method, and identified some different combinations of SNPs with prediction rates of 70.9% and 70.6%, respectively. For more precise identification of gene-gene interaction information in a biological manner, we may need more precise well-characterized sub-population datasets. In order to refine the genetic-environment relationship, more information is required in an epidemiological investigation. Besides existing statistical methods, we demonstrated the feasibility of incorporating SVM - a machine learning algorithm into case-control study.

## Results

### Case-Control Association Study

**Figure 1:**
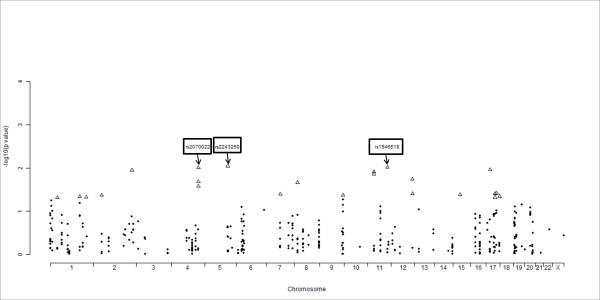
**Chromosome distribution for association of SNPs with Type 2 Diabetes**. In this panel, Manhattan plot shows distributed variables that were generated by genome-wide significance (p-values). Triangle markers at each locus indicate the significant SNPs based on a chi-squared test (p value < 0.05). Boxed SNPs represent Top 3 ranked lists.

For each SNP, the *p*-value was calculated based on a chi-squared test. Based on the test results of 408 SNPs, 27 SNPs showed a significant genotype- or allele-based *p*-value (< 0.05) (Table 1). The -log10 *p*-value from association result of SNPs was plotted in each chromosome and the significant SNPs are circle shape (Figure 1).

This candidate-gene based analysis may have some limitations to detect association from the small population size (462 cases and 456 controls) and the limited number of candidate genes (87 putative T2D-related genes). The result of this classical case-control association study may need a further replication study with a large independent target population of cases and controls for establishing the credibility of a genotype-phenotype association. We used this classical association study result in the process of sub-dataset filtering based on the genotype-based *p*-value range (Table 2).

**Table 1:** Summary of the association study (genotype- or allele-based p < 0.05)

Gene	dbSNP ID(b129)	Chromosome	Location	Region*	Alleles*	χ^2^p-value	
						
						Genotype	Allele
SELE	rs4786	1	167958756	3'UTR	G>A	0.0462	0.1299
IL10	rs1554286	1	205010856	intron	T>C	0.0470	0.0170
CAP1	rs16837478	1	40207033	3'UTR	C>A	0.0489	0.5850
VAMP3	rs707457	1	7753651	nearGene-5	G>T	0.0755	0.0299
VAMP3	novel	1	7775035	3'UTR	A>G	0.0567	0.0220
SLC11A1	novel	2	219084963	exon	G>A	0.0113	0.0229
RHOQ	rs17038378	2	46661749	nearGene-3	W>D	0.0426	0.1773
FGA	rs2070022	4	155724398	3'UTR	C>T	0.0099	0.0083
FGA	rs6050	4	155727040	exon	A>G	0.0261	0.0077
FGA	rs2070011	4	155731347	nearGene-3	A>G	0.0208	0.0053
IL4	rs2243250	5	132037053	nearGene-5	T>C	0.0090	0.0025
SOD2	rs5746136	6	160023074	intron	G>A	0.0948	0.0348
CD36	rs3211908	7	80131852	intron	C>T	0.0402	0.1445
LPL	rs343	8	19855067	intron	C>A	0.0215	0.0075
RAPGER1	rs875968	9	133461085	intron	G>A	0.0423	0.1247
IL18	rs1946518	11	111540668	nearGene-5	T>G	0.0097	0.1591
CAT	rs17886119	11	34417280	intron	C>T	0.0138	0.2849
CAT	rs1408034	11	34432364	intron	C>T	0.0123	0.2745
TCF1	rs1169288	12	119901033	exon	T>G	0.0391	0.6975
TCF1	rs2464196	12	119919810	exon	T>C	0.0181	0.9747
SNAP23	rs9302112	15	40607743	intron	T>C	0.0414	0.0842
ACE	rs4362	17	58927493	exon	C>T	0.0384	0.0270
NOS2A	rs2297518	17	23120724	exon	G>A	0.0109	0.5354
STXBP4	rs9902718	17	50416621	intron	T>C	0.0478	0.0156
STXBP4	rs10468513	17	50417902	intron	C>A	0.0478	0.0156
STXBP4	rs11658717	17	50431985	intron	A>G	0.0396	0.0128
ASPSCR1	novel	17	77562902	intron	G>T	0.0456	0.0511

* Alleles and region for which the effect is estimated refer to the positive strand based on NCBI build 36

**Table 2:** Prediction rate of combinations of SNPs with genotype-based p-value filtering

p-value range	**No. of SNPs **[1]	Prediction rate			**No. of SNPs **[2]
			
		Overall	Sensitivity	Specificity	
< 0.05	24	0.576	0.545	0.607	4 SNPs
< 0.1	40	0.600	0.593	0.607	6 SNPs
< 0.2	92	0.632	0.660	0.603	10 SNPs
< 0.3	129	0.642	0.630	0.654	12 SNPs
< 0.4	169	0.642	0.630	0.654	12 SNPs
< 0.5	199	0.651	0.571	0.732	13 SNPs
**< 0.6**	**240**	**0.653**	**0.567**	**0.739**	**14 SNPs**
< 0.7	290	0.651	0.610	0.693	12 SNPs
< 0.8	335	0.636	0.721	0.550	7 SNPs
< 0.9	372	0.636	0.721	0.550	7 SNPs
**<**1.0	408	0.636	0.721	0.550	7 SNPs

[1] No. of SNPs for each genotype-based *p*-value range; [2] No. of SNPs for each combination

### Combination of SNPs

We performed SVM training and test analysis to find the best combination of SNPs. The prediction rates were determined by the SVM classifier that discriminated the case-control SNP genotype vectors. At first, we acquired 63.6% of the overall accuracy with the entire 408 SNP dataset, but we found that the p-value-based filtering method is useful for obtaining a better prediction rate. The prediction rate of a higher *p*-value SNP dataset (Table 1) did not show the best result (57.6%). This effect might be attributed to the different effects between a single SNP and within a combination of SNPs. This *p*-value-based filtering can reduce the search space for gene-gene interactions from a very large number of all possible combinations of SNPs to a manageable dataset.

Another reason is the limitation of the forward selection method to find the best combination of SNPs. The entire set of 408 SNPs may contain noise SNPs for forward selection, and some useful SNPs in the ideal combination may be removed from the very restricted *p*-value-based filtered SNP dataset (e.g., 24 SNPs with *p *< 0.05).

The best prediction rate of the SVM classifier with a RBF kernel function was 65.3% with 14 SNPs including a combination from the 240 SNPs with p < 0.6 (Table 2 and Table 3). In table 3, rs343 was reported the association with T2D [35], and two of SNPs (rs2070011 and rs2243250) were reported with not T2D but myocardial infarction [36,37]. Furthermore, sub-population datasets of men and women with the RBF kernel, which were designed to discriminate case and control, yielded slightly better prediction rates of 70.9% and 70.6%, respectively, than that of the total population dataset (Table 4, Table 5 and Table 6). These prediction rates are almost similar with other previous studies using SVM, for example 69% of Listgarten and colleagues [33], 67.5% of Uhmn *et al*. [34], or 53% of Schwender *et al*. [38]. But, these previous works used different disease samples and different cross-validation test, thus it is difficult to compare these prediction rates directly. Considering other environmental and genetic factors involved in the development of T2D, the prediction performance was reasonably acceptable. It may be presumed that including other important genes and clinical factors including family medical history, we would obtain more improved prediction rate in the future. Different results between the entire target population and men or women sub-population may arise from the effect of the dataset's size or the well-characterized sub-population grouping.

**Table 3:** List of 14 SNPs for the best combination of SNPs

Gene	dbSNP ID	Chromosome	Location	Region	Allele
IRS1	rs6436635	2	227373922	nearGene-5	G>A
SLC11A1	Novel*	2	17459455	exon	G>A
FGA	rs2070011**	4	155731347	5'UTR	A>G
SPP1	rs2853749^#^	4	89116838	intron	C>T
IL4	rs2243250	5	132037053	nearGene-5	T>C
IL4	rs56279116	5	132038071	exon	G>A
PPARD	rs9658173	6	35502649	3'UTR	G>A
LPL	rs343^##^	8	19855067	intron	C>A
TCF1	rs2464196	12	119919810	exon	T>G
ACE	rs13306087	17	58910142	exon	G>A
ASPSCR1	Novel*	17	77562902	intron	G>T
NOS2A	rs9282801	17	23120600	intron	G>T
INSR	rs2303672	19	7119405	intron	A>G
INSR	rs3745548	19	7103703	intron	A>G

* Novel SNP in KHGS does not exist on dbSNP database.

** rs2070011 is associated with myocardial infarction [36]

^#^rs2243250 is associated with myocardial infarction [37]

^##^rs343 is associated with T2D [44]

**Table 4:** Prediction rates of the SVM classifiers with different target populations

Target population	Sensitivity	Specificity	Overall accuracy	No. of SNPs for each combination
Total	0.567	0.739	0.653	14 SNPs
Men^#^	0.714	0.704	0.709	12 SNPs
Women^##^	0.715	0.696	0.706	19 SNPs

^# ^199 Cases; 206 Controls

^## ^263 Cases; 250 Controls

**Table 5:** List of 12 SNPs for the best combination of SNPs (men)

Gene	dbSNP ID	Chromosome	Location	Region	Allele
LEPR	rs1805134	1	65839697	exon	A>G
PRKCZ	rs3795277	1	1970978	nearGene-5	A>C
PPARG	rs13306747	3	12433274	exon	C>G
FABP2	rs1799883	4	120461350	exon	G>A
UCP1	novel	4	141848403	promoter	G>A
IL4	novel	5	132038071	exon	G>A
LPL	rs3208305	8	19867928	3'UTR	A>T
LPL	rs13702	8	19868772	3'UTR	A>G
STXBP4	rs1894936	17	50475854	intron	A>G
LDLR	rs6413504	19	11102915	intron	A>G
LDLR	rs1433099	19	11103658	3'UTR	G>A
ACAS2	novel	20	32926612	promoter	G>T

**Table 6:** List of 19 SNPs for the best combination of SNPs (women)

Gene	dbSNP ID	Chromosome	Location	Region	Allele
CAP1	rs16837478	1	40207033	3'UTR	C>A
IL10	rs1554286	1	203332628	intron	T>C
LEPR	rs13306523	1	65603011	5'UTR	C>T
MTHFR	rs2274976	1	11785193	exon, nearGene-3	G>A
SELE	rs5355	1	166427528	exon	C>T
VAMP3	novel	1	7775035	3'UTR	A>G
IRS1	rs6436635	2	227373922	nearGene-5	G>A
IRS1	rs1801278	2	227486049	exon	G>A
NEUROD1	rs1801262	2	182368961	exon	G>A
SLC11A1	novel	2	219084963	exon	G>A
GPX1	rs3811699	3	49371364	nearGene-5	A>G
IL4	novel	5	132038071	exon	G>A
SLC27A4	rs17848327	9	128192130	intron	G>A
MMP2	rs17859889	16	54077202	intron	C>T
MMP2	rs17860021	16	54097255	3'UTR	G>A
ASPSCR1	novel	17	77528191	nearGene-5	G>C
STXBP4	rs10468513	17	50417902	nearGene-5	C>A
STXBP4	rs11658717	17	50431985	intron	A>G
INSR	novel	19	7135243	intron	G>C

We could not find better prediction results by the above *p*-value-based filtering as that in Table 2 with men and women sub-population datasets. This result with a slightly improved prediction rate may arise from the effect of a smaller size of sub-datasets (n = 405 and 513) or the effect of well-characterized (gender-distinguished) sub-population datasets.

### Protein-Protein Interaction Information

**Figure 2:**
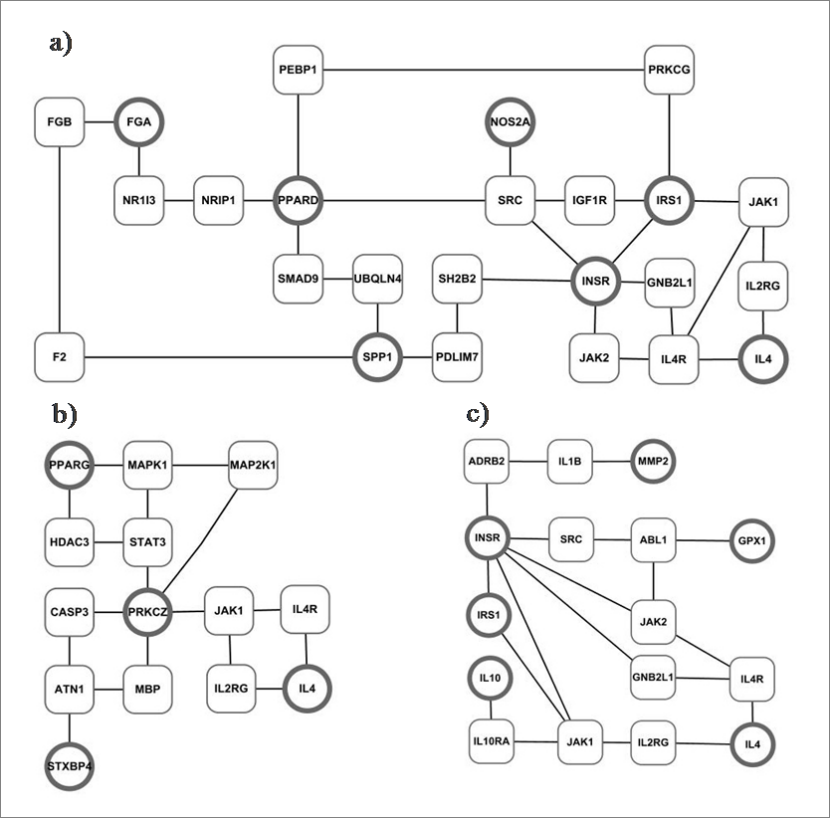
**PPI network from the SNP combination of (a) total population set, (b) men sub-population set, and (c) women sub-population set**. The largest PPI network at each population set was constructed using PPI information database http://genomenetwork.nig.ac.jp. Circles are included in the best combination of SNPs from SVM results and square proteins are included to construct circle-circle connected proteins network.

On the basis of the results of the combinations of SNPs, we attempted to find any biological information; one of the results is the protein-protein interaction (PPI) network (Figure 2), which was constructed from the results of the combinations of SNPs. Each set of the SNP genotype data was not acquired from the fine mapping association study; therefore, direct SNP-SNP interaction or SNP analysis focused on each promoter SNP, intron SNP, or exon SNP is difficult. This is the reason why we carried out the analysis at the protein (gene) level in this research (not the SNP level).

The genomenetwork platform http://genomenetwork.nig.ac.jp provides protein-protein interaction network from the Y2H experimental data and the public databases (BIND, MINT and HPRD). Also, it has interaction property and gene annotation information. We obtained gene interaction information from PPI database. Circles (proteins) are included in the results of the combination of SNPs, and circles are collected from the entire PPI information database to connect with the squares. The construction of an indirect PPI network of two proteins is unnecessary from the biological viewpoint; therefore, we permit only two or fewer proteins (squares) between two proteins (circles) in Figure 2. We could easily find the same proteins among the target population datasets and the target-population-specific proteins in these three PPI networks. PPI network of Figure 2a contains 7 genes from the SNP combination result of 12 genes (14 SNPs) in Table 3. Other PPI network of Figure 2b and 2c contains 4 genes and 6 genes from the SNP combination results of men and women sub-data sets in Table 5 and 6, respectively. *IL4 *(interleukin 4) gene is the common gene among these three PPI networks and *IL4*, *INSR*, and *IRS1 *genes are common between Figure 2a (total population set) and 2c (women sub-population set).

## Discussion

It is widely agreed that complex diseases are typically caused by the joint effects of multiple genetic variations instead of a single genetic variation. The gene-gene (epistatic) interactions of SNPs are believed to be very important in determining individual susceptibility to complex diseases. Thus, it is desirable to develop an effective method to search gene-gene interactions in human genome data. Recently, some computational methods have been proposed to address this issue using Multifactor Dimensional Reduction (MDR), or machine learning algorithms [39]. To study complex disease such as T2D, it is possible that many genes contribute to a T2D by their interaction with other genes, while main effects of the individual gene may be small or absent. Therefore, we developed the method that specifically designed to detect multiple disease SNPs, possibly on different chromosomes using SVM. This approach could be useful for identification of potential disease markers which genotype patterns are significantly associated with a high susceptibility.

This analysis includes the SNPs information of 87 T2D-related genes from fatty acid binding/translocation, GLUT4 translocation, and insulin signal pathways. A primary function of insulin is to stimulate the transport of glucose into target tissue, prominent among which are skeletal muscle, cardiac muscle, and adipose tissue. Insulin achieves this effect by inducing the translocation of GLUT4 glucose transporters from an intracellular vesicular compartment to the plasma membrane. Under basal condition, GLUT4 cycles between this intracellular compartment and the plasma membrane. SNAP23 is required for insulin-induced GLUT4 translocation to the plasma membrane and that it mediates the formation of a complex between syntaxin4 and VAMP2 [40].

T2D results from impairment in both insulin sensitivity and insulin secretion. Several genes have been implicated that might contribute significantly to the risk of T2D, including *TCF7L2, PPARG, KCNJ11, CDKN2A/B *and so on [6,8-10]. T2D is one of the typical complex disease (polygenic disorder), which likely associated with the effects of multiple genes (SNPs) in combination with lifestyle and other environmental factors. In this research, we analyzed the candidate genes data set, thus the result does not contain these known significant SNP markers, including *TCF7L2, PPARG, KCNJ11 *and so on.

The result from Table 1 (all cases, combined versus controls) indicated weak associations with the risk factors investigated. This led us to stratify by sub-grouping by gender to see whether some potential associations may have been obscured by considering T2D as one disease. In this research, we first made two subpopulation data sets by gender (Table 4, and Figure 2). Epidemiological evidence suggests that sex differences exist in T2D. The prevalence of T2D is higher in men than women. Globally, diabetes prevalence is similar in men and women but it is slightly higher in men < 60 years of age and in women at older ages [1]. This difference may possibly result from the differences in insulin sensitivity and regional body fat deposition [41,42].

Yeh *et al*. [43] used a conditional knockout strategy to generate androgen receptor (AR) knockout mice to study the relationship between androgen-AR and insulin sensitivity, and Lin et al. reported the influences of loss of AR on insulin and leptin resistance. Loss of AR may contribute to an increase of leptin levels and leptin resistance, which may play important roles for the development of obesity and insulin resistance. Important factors such as age at onset of T2D can also be incorporated in the modeling to further partition phenotypic variation or for defining subtypes of the phenotype.

As high-throughput technology for genome-wide SNP genotyping (500 K or 1 mega) improves and as more SNPs are identified, it is likely that much higher prediction rate will be achieved and a useful clinical system developed. For the biologically more precise identification of gene-gene interaction's effect for T2D, we may need more precise well characterized subpopulation data sets and more powerful computational power and method and so on. Besides existing statistical methods, we demonstrated the feasibility of incorporating SVM - a machine learning algorithm into case-control study. We plan to develop the method using machine learning algorithm in the future to search gene-gene interactions for our new Genome-Wide Association Study (GWAS) data [44].

## Conclusions

We have found novel association between combinations of SNPs and T2D in a Korean population. We proposed gene-gene interaction considering candidate genes association study using SVM based feature selection method in this research.

## Methods

### Data and Data Preprocessing

Our dataset consists of 408 SNP data distributed over putative 87 T2D-related genes in 462 cases (patients) and 456 normal controls. The T2D cases, confirmed and diagnosed in the Ansan and Ansung cohort study area, were identified from the Korean Health and Genome Study (KHGS). The Ansan area primary represents an urban community, whereas the Ansung area represents a rural community in Korea. These two cohort studies include information on 87 T2D-related genes from fatty acid binding/translocation, GLUT4 (insulin-responsive glucose transporter 4) translocation, and insulin signal pathways. We selected these three pathways with reference survey [45-48]. Among identified polymorphisms, 408 SNPs were selected based on location (CDS (protein coding sequence), intron, UTR (Untranslated region), promoter etc), frequency, linkage disequilibrium (LD) status and so on. The number of SNPs occurring in the CDS, intron, UTR, near gene region (promoter) and intergenic region was 77, 169, 53, 73 and 36, respectively (additional file 1). In this research, 462 cases were defined from two cohort studies as T2D subjects according to the World Health Organization (WHO) criteria. The 456 unrelated normal control people have no history of T2D, no first-degree relatives with T2D, fasting plasma glucose level less than 126 mg/dL, plasma glucose level 120 min after glucose ingestion of less than 140 mg/dL, and HbA1C level (glycosylated hemoglobin) of less than 5.8%. Further, the normal control people do not have a history of diabetes, hypertension, and dyslipidemia. In this study, all the people of case and control were more than 60 years of age.

For each SNP, the *p*-value was calculated based on a chi-squared test without adjustment for other confounding variables (Table 1). In this paper, we applied SVM to predict the susceptibility to T2D using SNP genotype data. From the view point of binary classification, we treated T2D cases as positive samples and controls as negative samples, and we used SNP variants as categorical features that have three possible genotype values at a locus. Usually, a SNP genotype is represented by a number that matches 1, 2, or 3, where 1 represents the homozygous site with a major allele, 2 represents a heterozygous site, and 3 represents a homozygous site with a minor allele [33]. Waddell *et al*. (2005) have applied SVMs to predict the susceptibility to multiple myeloma using -1, 0, 1, where 0 represents a heterozygous site and -1 and 1 arbitrarily represent homozygous sites. The preprocessing method used in this research was the same as that used by Listgarten *et al*. (2004) [33].

### Support Vector Machine

A SVM is a learning algorithm that learns a classifier from a set of positively and negatively labeled training vectors, which can be used to classify new unlabelled test samples. The SVM learns the classifier by mapping the input training samples into a possibly high-dimensional feature space, and seeking a hyperplane in this space that separates the two types of examples with the largest possible margin, i.e., the distance to the nearest points. If the training set is not linearly separable, the SVM finds a hyperplane that optimizes the trade-off between good classification and large margin with a slack variable and kernel trick. For an actual implementation, we used the freely downloadable SVM-light package [49]. We tested linear, polynomial, and radial basis function (RBF) kernels with various parameters, and the final results were acquired with the RBF kernel and parameter gamma 1 that yielded the best prediction rate. We treated T2D cases as positive samples and controls as negative samples, and used SNP genotypes as categorical features. We adopted SVM to discriminate T2D cases against controls in this research.

### Feature Selection

For large datasets, an exhaustive consideration of all the possible SNP combinations can become computationally infeasible. Therefore, we employed a feature selection procedure to find the best putative combination of SNPs according to forward selection for handling datasets with a genotype of SNPs. In population studies, this kind of selection of informative SNPs was usually developed for population identification [50]. In this work, forward selection was started by first selecting the SNP feature that yielded the best fit for the independent test set using SVM training and test at a time. This SNP feature was used to test all the combinations with the remaining 407 (408 - 1) SNPs in order to find the best pair of SNP features. This process continues step by step until increasing the size of the current subset leads to a lower overall accuracy. We adopted the 10-fold cross-validated classification accuracy for the selection criteria in this work. The requirement of the best prediction rate at each step yields the highest overall accuracy with regard to both sensitivity and specificity (≥ 0.45). The purpose of this requirement is to avoid the extremely low sensitivity or specificity with the highest overall accuracy.

Since we have a relatively small number of people (462 cases and 456 controls) in our dataset, it is expected that training with the complete set of 408 SNP features may cause overfitting. Hence, we performed forward selection with SNP genotype features to find a good smaller feature set (a combination of SNPs). Note that forward selection does not necessarily find the best combination of SNPs. However, it usually results in a combination that comes close to the optimum solution, and it needs relatively less computational complexity. If we have a smaller datasets and a more powerful computer, step-wise feature selection may be a better method than the forward selection method in this study.

### Cross-Validation Test

The prediction rates of the SVM classifiers were examined by the 10-fold cross-validation test, where each case and control dataset is randomly divided into 10 subsets of approximately the same size. The SVM classifiers were trained 10 times, leaving out one of the subsets from the training each time. This single subset was used to estimate the prediction rate of the trained SVM classifier. The prediction rate of the SVM classifiers was evaluated using three measures, namely, sensitivity, specificity, and overall accuracy.

where *TP*, *FP*, *TN*, and *FN *refer to the number of true positives, false positives, true negatives, and false negatives statuses (case or control), respectively. Sensitivity measures the ability to correctly predict T2D cases, while specificity measures the ability for correctly reject controls. The kernel functions and parameters for the classification algorithms were optimized during the 10-fold cross-validation tests, while avoiding overfitting problems.

## Authors' contributions

HJB drafted the manuscript, participated in the design the study, and contributed to the statistical and functional analysis. JYH co-designed the study, performed the SVM machine learning and statistical analysis, KSO helped draft the manuscript and critically reviewed the manuscript. KJP investigated and guided the study project and drafted the manuscript. All authors read and approved the final manuscript.

## Supplementary Material

Additional file 1 **General information of 408 SNPs data set**. 408 SNPs were selected 87 T2D-related genes from fatty acid binding/translocation, GLUT4 (insulin-responsive glucose transporter 4) translocation and insulin signal pathways. Excel file shows the general SNP information including SNP ID (rs number), chromosomal location information and so on. Click here for file

## References

[B1] Wild S Roglic G Green A Sicree R King H Global prevalence of diabetes: estimates for the year 2000 and projections for 2030 Diabetes Care 2004 27 1047 1053 10.2337/diacare.27.5.1047 15111519

[B2] Association AD Economic consequences of diabetes mellitus in the U.S. in 1997. American Diabetes Association Diabetes Care 1998 21 296 309 10.2337/diacare.21.2.296 9539999

[B3] Owen KR McCarthy MI Genetics of type 2 diabetes Curr Opin Genet Dev 2007 17 3 239 244 10.1016/j.gde.2007.04.003 17466512

[B4] Hunter DJ Kraft P Drinking from the fire hose - statistical issues in genome wide association studies N Engl J Med 2007 357 436 439 10.1056/NEJMp078120 17634446

[B5] Kraft P Hunter DJ Genetic Risk Prediction-Are We There Yet? N Engl J Med 2009 360 17 1701 1703 10.1056/NEJMp0810107 19369656

[B6] Altshuler D Hirschhorn JK Klannemark M Lindgren CM Vohl MC Nemesh J Lane CR Schaffner SF Bolk S Brewer C The common PPARgamma Pro12Ala polymorphism is associated with decreased risk of type 2 diabetes Nat Genet 2000 26 1 76 80 10.1038/79216 10973253

[B7] Consortium WTCC Genome-wide association study of 14,000 cases of seven common diseases and 3,000 shared controls Nature 2007 447 661 678 10.1038/nature05911 17554300 PMC2719288

[B8] Gloyn AL Weedon MN Owen KR Turner MJ Knight BA Hitman G Walker M Levy JC Sampson M Halford S Large-scale association studies of variants in genes encoding the pancreatic beta-cell KATP channel subunits Kir6.2 (KCNJ11) and SUR1 (ABCC8) confirm that the KCNJ11 E23K variant is associated with type 2 diabetes Diabetes 2003 52 2 568 572 10.2337/diabetes.52.2.568 12540637

[B9] Grant SF Thorleifsson G Reynisdottir I Benediktsson R Manolescu A Sainz J Helgason A Stefansson H Emilsson V Helgadottir A Variant of transcription factor 7-like 2 (TCF7L2) gene confers risk of type 2 diabetes Nat Genet 2006 38 3 320 323 10.1038/ng1732 16415884

[B10] Scott LJ Mohlke KL Bonnycastle LL Willer CJ Li Y Duren WL Erdos MR Stringham HM Chines PS Jackson AU Prokunina-Olsson L A genome-wide association study of type 2 diabetes in Finns detects multiple susceptibility variants Science 2007 316 5829 1341 1345 10.1126/science.1142382 17463248 PMC3214617

[B11] Masher B Personal genomes: The case of the missing heritability Nature 2008 456 18 21 10.1038/456018a 18987709

[B12] Hardy J Singleton A Genomewide Association Studies and Human Disease N Engl J Med 2009 360 1759 1768 10.1056/NEJMra0808700 19369657 PMC3422859

[B13] Jakobsdottir J Gorin MB Conley YP Ferrell RE Weeks DE Interpretation of genetic association studies: markers with replicated highly significant odds ratios may be poor classifiers PLos Genet 2009 5 2 e1000337 10.1371/journal.pgen.1000337 19197355 PMC2629574

[B14] Hahn LW Ritchie MD Moore JH Multifactor dimensionality reduction software for detecting gene-gene and gene-environment interactions Bioinformatics 2003 19 376 382 10.1093/bioinformatics/btf869 12584123

[B15] Goodman JE Mechanic LE Luke BT Ambs S Chanock S Harris CC Exploring SNP-SNP interactions and colon cancer risk using polymorphism interaction analysis Int J Cancer 2006 118 7 1790 1797 10.1002/ijc.21523 16217767 PMC1451415

[B16] Kecman V Learning and Soft Computing, Support Vector machines, Neural Networks and Fuzzy Logic Models 2001 The MIT Press, Cambridge, MA

[B17] Khandoker AH Support Vector Machines for Automated Recognition of Obstructive Sleep Apnea Syndrome from ECG Recordings IEEE Trans Inf Technol Biomed 2009 13 1 37 48 10.1109/TITB.2008.2004495 19129022

[B18] Sloin A Burshtein D Support Vector Machine Training for Improved Hidden Markov Modeling IEEE trans signal process 2008 56 1 172 10.1109/TSP.2007.906741

[B19] Wang LP Support Vector Machines: Theory and Application 2005 Springer, Berlin

[B20] Wang LP Fu XJ Data Mining with Computational Intelligence 2005 Springer, Berlin

[B21] Nguyen MN Rajapakse JC Two-stage multi-class support vector machines to protein secondary structure prediction Pac Symp Biocomput 2005 10 346 357 full_text 10.1142/9789812702456_0033 15759640

[B22] Yuan Z Burrage K Mattick JS Prediction of protein solvent accessibility using support vector machines Proteins 2002 48 566 570 10.1002/prot.10176 12112679

[B23] Kim H Park H Prediction of protein relative solvent accessibility with support vector machines and long-range interaction 3D local descriptor Proteins 2004 54 3 557 562 10.1002/prot.10602 14748002

[B24] Bradford JR Westhead DR Improved prediction of protein-protein binding sites using a support vector machines approach Bioinformatics 2005 21 1487 1494 10.1093/bioinformatics/bti242 15613384

[B25] Busuttil S Abela J Pace GJ Support vector machines with profile-based kernels for remote protein homology detection Genome Inform 2004 15 2 191 200 15706505

[B26] Wang C Ding C Meraz RF Holbrook SR PSoL: A positive sample only learning algorithm for finding non-coding RNA genes Bioinformatics 2006 22 2590 2596 10.1093/bioinformatics/btl441 16945945

[B27] Vlahovicek K Kajan L Agoston V Pongor S The SBASE domain sequence resource, release 12: Prediction of protein domain-architecture using support vector machines Nucleic Acids Res 2005 33 D223 225 10.1093/nar/gki112 15608182 PMC540066

[B28] Hua S Sun Z Support vector machine approach for protein subcellular localization prediction Bioinformatics 2001 17 721 728 10.1093/bioinformatics/17.8.721 11524373

[B29] Nair R Rost B Mimicking cellular sorting improves prediction of subcellular localization J Mol Biol 2005 348 85 100 10.1016/j.jmb.2005.02.025 15808855

[B30] Park K-J Kanehisa M Prediction of protein subcellular locations by support vector machines using compositions of amino acid pairs Bioinformatics 2003 19 1656 1663 10.1093/bioinformatics/btg222 12967962

[B31] Park K-J Gromiha MM Horton P Suwa M Discrimination of outer membrane proteins using support vector machines Bioinformatics 2005 21 23 4223 4229 10.1093/bioinformatics/bti697 16204348

[B32] Brown MPS Grundy WN Lin D Cristianini N Sugnet CW Furey TS Ares M Haussler D Knowledge-based analysis of microarray gene expression data by using support vector machines Proc Natl Acad Sci USA 2000 97 262 267 10.1073/pnas.97.1.262 10618406 PMC26651

[B33] Listgarten J Damaraju S Poulin B Cook L Dufour J Driga A Mackey J Wishart D Greiner R Zanke B Predictive models for breast cancer susceptibility from multiple single nucleotide polymorphism Clin Cancer Res 2004 10 2725 2737 10.1158/1078-0432.CCR-1115-03 15102677

[B34] Uhmn S Kim D-H Ko Y-W Cho S Cheong J Kim J A study on application of single nucleotide polymorphism and machine learning techniques to diagnosis of chronic hepatitis Expert Systems 2009 26 60 69 10.1111/j.1468-0394.2008.00491.x

[B35] Cho YS Go MJ Han HR Cha SH Kim HT Min H Shin HD Park C Han BG Cho NH Association of lipoprotein lipase (LPL) single nucleotide polymorphisms with type 2 diabetes mellitus Exp Mol Med 2008 40 5 523 532 10.3858/emm.2008.40.5.523 18985010 PMC2679352

[B36] Mannila MN Lovely RS Kazmierczak SC Eriksson P Samnegård A Farrell D Hamsten A Silveira A Elevated plasma fibrinogen gamma' concentration is associated with myocardial infarction: effects of variation in fibrinogen genes and environmental factors J Thromb Haemost 2007 5 4 766 773 10.1111/j.1538-7836.2007.02406.x 17263791

[B37] Paffen E Medina P de Visser M van Wijngaarden A Zorio E Estellés A Rosendaal FR España F Bertina RM Doggen CJ The -589C>T polymorphism in the interleukin-4 gene (IL-4) is associated with a reduced risk of myocardial infarction in young individuals J Thromb Haemost 2008 6 10 1633 1638 10.1111/j.1538-7836.2008.03096.x 18662263

[B38] Schwender H Zucknick M Ickstadt K Hermann MB network TG A Pilot study on the application of statistical classification procedures to molecular epidemiological datatoxicology letters 2004 151 291 299 10.1016/j.toxlet.2004.02.021 15177665

[B39] Musani SK Shriner D Liu N Feng R Coffey CS Yi N Tiwari HK Allison DB Detection of gene x gene interactions in genome-wide association studies of human population data Hum Hered 2007 63 2 67 84 10.1159/000099179 17283436

[B40] Kawanishi M Tamori Y Okazawa H Araki S Shinoda H Kasuga M Role of SNAP23 in insulin-induced translocation of GLUT4 in 3T3-L1 adipocytes. Mediation of complex formation between syntaxin4 and VAMP2 J Biol Chem 2000 275 8240 8247 10.1074/jbc.275.11.8240 10713150

[B41] Gale EA Gillespie KM Diabetes and gender Diabetologia 2001 44 1 3 15 10.1007/s001250051573 11206408

[B42] Lin HY Xu Q Yeh S Wang RS Sparks JD Chang C Insulin and leptin resistance with hyperleptinemia in mice lacking androgen receptor Diabetes 2005 54 1717 1725 10.2337/diabetes.54.6.1717 15919793

[B43] Yeh S Hu YC Wang PH Xie C Xu Q Tsai MY Dong Z Wang RS Lee TH Chang C Abnormal mammary gland development and growth retardation in female mice and MCF7 breast cancer cells lacking androgen receptor J Exp Med 2003 198 1899 1908 10.1084/jem.20031233 14676301 PMC2194158

[B44] Cho YS Go MJ Kim YJ Heo JY Oh JH Ban HJ Yoon D Lee MH Kim DJ Park M A large-scale genome-wide association study of Asian populations uncovers genetic factors influencing eight quantitative traits Nat Genet 2009 41 5 527 534 10.1038/ng.357 19396169

[B45] Vollenweider P Insulin resistant states and insulin signaling Clin Chem Lab Med 2003 41 9 1107 1119 10.1515/CCLM.2003.173 14598859

[B46] Chang L Chiang SH Saltiel AR Insulin signaling and the regulation of glucose transport Mol Med 2004 10 7-12 65 71 16307172 10.2119/2005-00029.Saltiel PMC1431367

[B47] Valverde AM Benito M Lorenzo M The brown adipose cell: a model for understanding the molecular mechanisms of insulin resistance Acta Physiol Scand 2005 183 1 59 73 10.1111/j.1365-201X.2004.01384.x 15654920

[B48] Delarue J Magnan C Free fatty acids and insulin resistance Curr Opin Clin Nutr Metab Care 2007 10 2 142 148 10.1097/MCO.0b013e328042ba90 17285001

[B49] Joachims T Making large-scale SVM learning practical. Advances in kernel methods - support vector learning 1999 MIT Press 169 184

[B50] Zhou N Wang L Effective selection of informative SNPs and classification on the HapMap genotype data BMC Bioinformatics 2007 8 484 10.1186/1471-2105-8-484 18093342 PMC2245981

